# Extended myectomy for apical hypertrophic cardiomyopathy: a case report

**DOI:** 10.1186/s13019-021-01745-y

**Published:** 2021-12-30

**Authors:** Daiki Saitoh, Mike Saji, Schuichiro Takanashi

**Affiliations:** 1 Department of Cardiovascular Surgery, Sakakibara Heart Institute, Tokyo, Japan; 2 Cardiology, Sakakibara Heart Institute, Tokyo, Japan

**Keywords:** Apical hypertrophic cardiomyopathy, Myectomy, Apical myectomy, Left ventricular end-diastolic volume, Apical cavity obliteration

## Abstract

**Background:**

Apical hypertrophic cardiomyopathy is a variant of hypertrophic cardiomyopathy that predominantly affects the apex of the left ventricle and rarely involves the right ventricular apex or both apexes. Heart transplantation is the traditional treatment for apical hypertrophic cardiomyopathy. Although surgical myectomy approaching the apex has been available for decades, its safety and accuracy greatly depend on the surgeon’s skills and experience.

**Case presentation:**

The first case involved a 63-year-old man with apical hypertrophic cardiomyopathy, wherein preoperative contrast computed tomography findings revealed apical hypertrophy and complete apical cavity obliteration. The patient underwent extended myectomy, which revealed the apex cavity filled with abnormal muscles. Using the transaortic approach, the location of the bilateral papillary muscle was confirmed, thereby providing the required orientation. The abnormal muscle mass was successfully resected, and the postoperative end-diastolic volume was extremely increased. The second case involved a 43-year-old man with an apical left ventricular aneurysm and mid-hypertrophic cardiomyopathy obstruction. The thin-walled apical aneurysm contained a large apical-basal band. Upon detecting the bilateral papillary muscle, mid-ventricular myectomy was performed from the apex. During postoperative catheterization, there was no pressure gradient between the left ventricle and aorta.

**Conclusions:**

We reviewed two cases of apical hypertrophic cardiomyopathy, efficiently treated using extended apical myectomy. Although it is an uncommon procedure, the cases presented show how it can be used to successfully manage cases of apical hypertrophic cardiomyopathy. However, it is important to secure the postoperative left ventricular end-diastolic volume.

## Background

Apical hypertrophic cardiomyopathy (aHCM) is a variant of HCM first described in 1976. It predominantly affects the left ventricular apex and rarely involves the right ventricular apex or both apexes. Furthermore, aHCM presents unique echocardiographic findings of asymmetrical apical hypertrophy in an ace-of-spades configuration [1, 2]. Approximately 40% of HCM cases involve aHCM. The condition is relatively uncommon in western countries and occurs more frequently in Asian populations [3–7]. aHCM is traditionally treated using heart transplantation. Surgical myectomy approaching the apex (apical myectomy) has been available for decades [8]; however, its safety and accuracy greatly depend on the surgeon’s skills and experience [9]. Herein, we report two cases of patients with aHCM who underwent extended myectomy.

## Case presentation

### Case 1

A 63-year-old man with an 8-year history of aHCM had remained healthy for 7 years until he was diagnosed with aHCM, during which he began to experience exertional dyspnea. He was referred to our hospital for surgical aHCM treatment. Preoperative contrast computed tomography (CT) revealed apical hypertrophy and complete apical cavity obliteration. Preoperative transesophageal echocardiography (TEE) revealed no systolic anterior motion (SAM) and an abnormal apical-basal band.

**Fig. 1:**
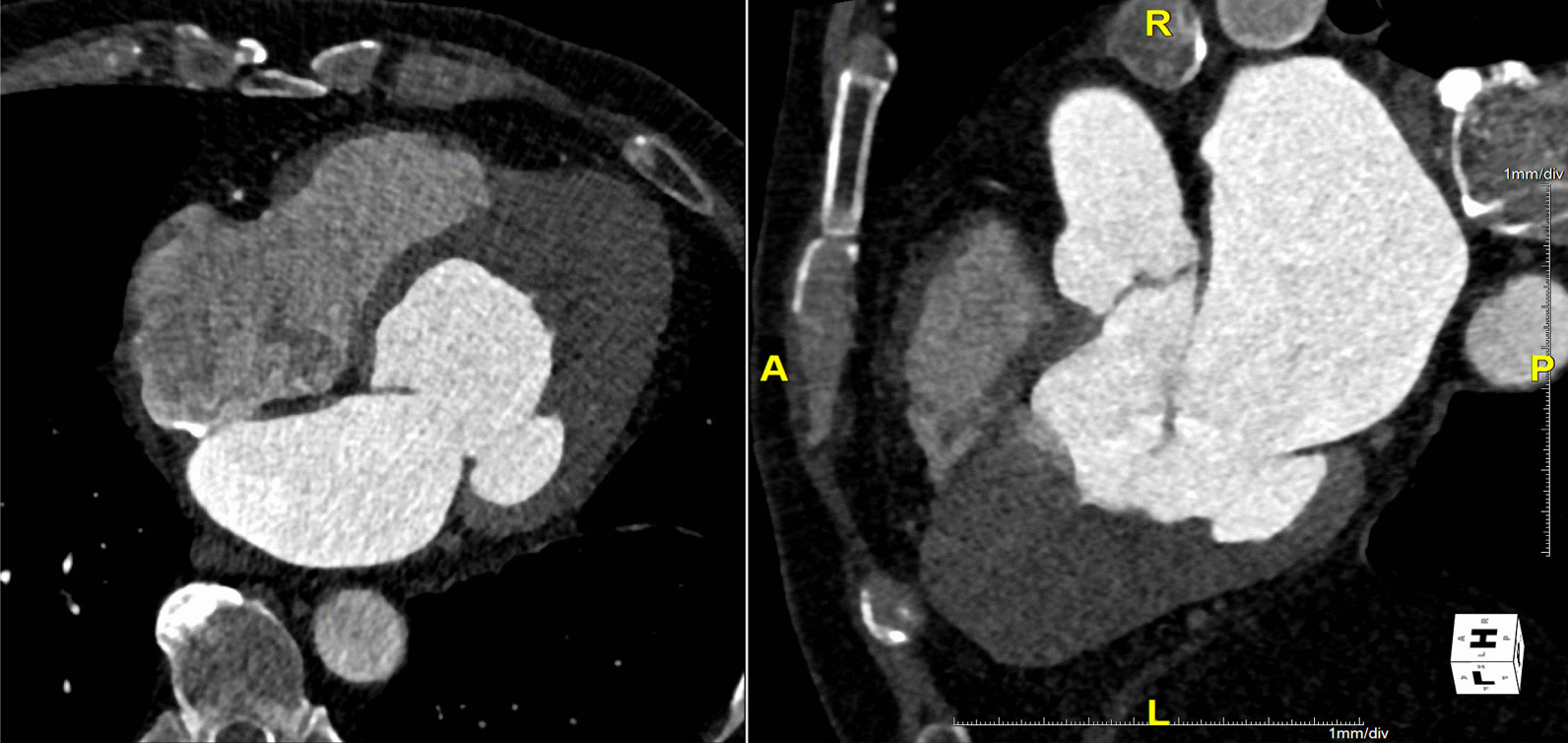
Preoperative CT images from Case 1. CT, computed tomography

**Fig. 2:**
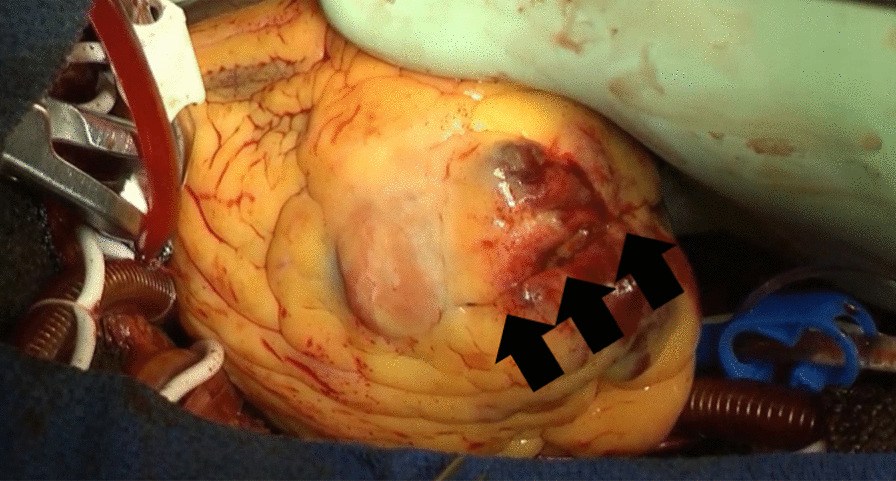
Intraoperative finding shows the incision line of the apex

**Fig. 3:**
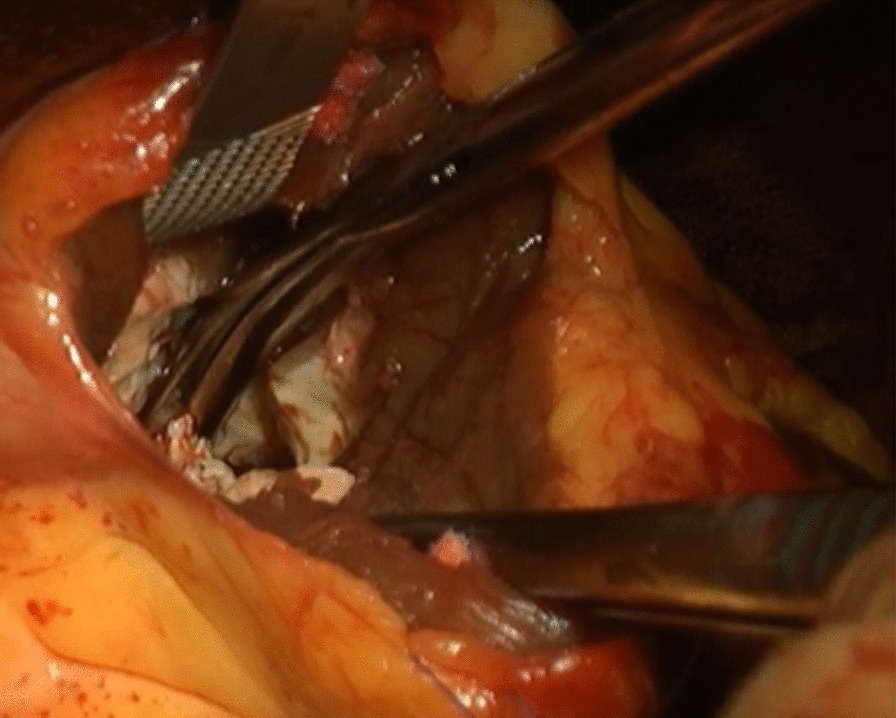
Intraoperative finding shows white fibrous endocardial muscles

**Fig. 4:**
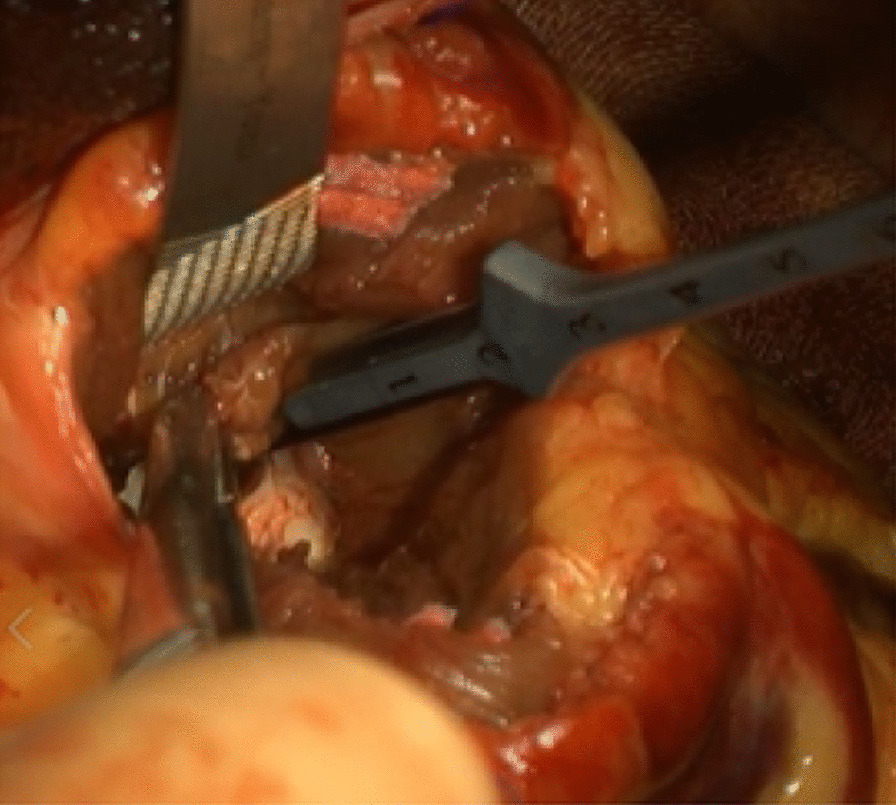
Intraoperative finding shows excess hypertrophic muscles on the LV free wall and abnormal papillary muscles are shaved. LV, left ventricular

The patient underwent extended myectomy, which revealed the apex cavity filled with abnormal muscles (Fig. 1). Therefore, we could not decide on the orientation for making an incision on the myocardium. The ventriculotomy line was created lateral and parallel to the left anterior descending artery. The incision line was over the apex, with a length of approximately 5–10 cm (Fig. 2). The cell saver sucker and cardiac sucker were used to retract the papillary muscles; consequently, additional incisions were made. When white fibrous endocardial muscles were identified, we used two 4–0 pledget monofilament sutures to retract the left ventricular (LV) wall on each side (Fig. 3). Excess hypertrophic muscles on the LV free wall and abnormal papillary muscles were shaved (Fig. 4). Using the transaortic approach, we confirmed the location of the bilateral papillary muscle, which indicated the required orientation. Initially, a bidirectional tunnel approach was performed between the bilateral papillary muscle and resected area to avoid the apex. We used 2–0 monofilament sutures for LV closure. The cardiopulmonary bypass time was 148 min, and the aortic clamp was on for 93 min.

**Fig. 5:**
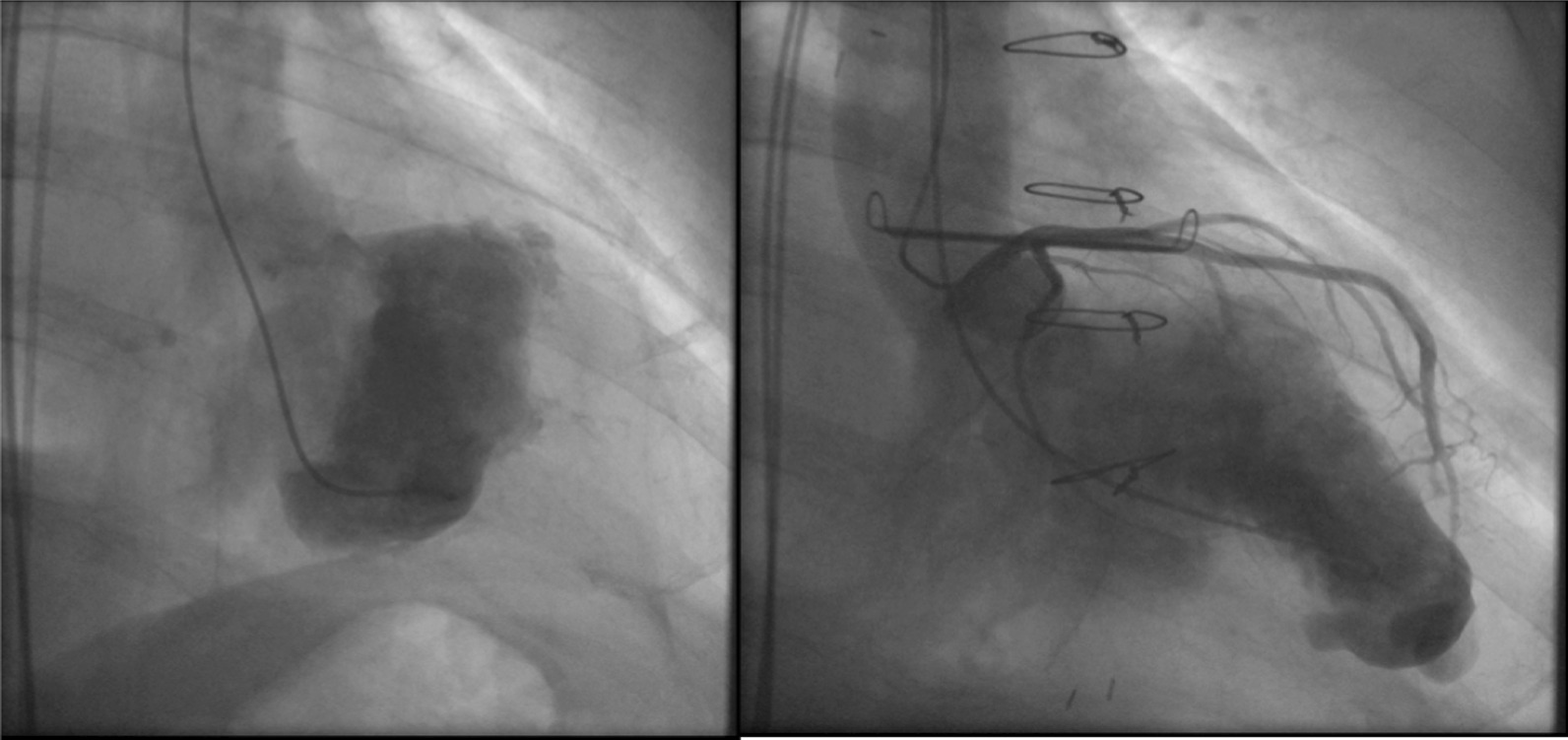
Pre- and postoperative cardiography images from Case 1

Postoperative contrast CT findings showed a small LV aneurysm, which was surgically resected 3 weeks after the initial myectomy. On postoperative day 3, the patient developed septic shock because of urinary tract infection and mediastinitis. After antibiotic administration and omentopexy, he was transferred to his previous hospital where he underwent 90 days of rehabilitation. Postoperative TEE demonstrated an LV end-diastolic volume (LVEDV)/end-systolic volume of 105.3/56.5 mL (preoperative, 67.2/35.9 mL). Postoperative left ventriculogram and angiography findings are presented in Fig. 5. The patient’s exertional dyspnea improved, and he was discharged 30 days after being transferred to the referring hospital.

### Case 2

A 43-year-old man with an apical LV aneurysm and mid-HCM obstruction was referred to our hospital for further evaluation. Eight years earlier, he was fitted with an implantable cardioverter-defibrillator (ICD) for non-sustained ventricular tachycardia. Mid-HCM was detected using CT and transthoracic echocardiography (TTE), and he was followed up as an outpatient for 5 years. One month before presenting at our hospital, he developed syncope and palpitations.

Preoperative contrast CT findings revealed an apical aneurysm and mid-ventricular obstruction. Preoperative TEE revealed mid-acceleration but no SAM. He underwent extended myectomy to alleviate palpitations and arrhythmia. A large apical-basal band was located inside the thin-walled apical aneurysm. Mid-ventricular myectomy was performed from the apex after detecting the bilateral papillary muscle. The resected myocardium weighed 9 g. The aneurysm was removed, and the incision was closed. Cardiopulmonary bypass lasted for 85 min, and the aorta was clamped for 50 min. Postoperative TEE findings revealed no SAM or ventricular septal perforation. During postoperative catheterization, there was no pressure gradient between the left ventricle and aorta.

On postoperative day 8, the patient exhibited atrial flutter, and defibrillation was performed via an ICD, which restored sinus rhythm. He was discharged on postoperative day 10.

## Discussion and conclusions

LV septal myectomy is the gold standard surgical treatment for LV outflow tract obstruction (LVOTO) in patients with HCM [10]. However, the safety and accuracy of apical myectomy depend on the surgeon’s skills and experience [9], as it is an uncommon procedure. Apical myectomy can be used for aHCM repair; however, postoperative LVEDV should be secured. Postoperatively, cardiac output likely improves because of increased outflow area and reduced drag forces on the anterior mitral leaflet (AML) by changing the flow vector in the outflow tract, which is more parallel to the AML.

The patient from Case 1 had extensive hypertrophy, especially in the apex; therefore, we could not perform myectomy using only the transaortic approach. However, using both apex and transaortic approaches, we were able to determine the positions of the bilateral papillary muscles, chordates, and mitral valve. This was our first case of treating aHCM using a bidirectional approach. In Case 2, extended myectomy was preferred because we considered that the removal of both the thin apex aneurysm and part of the septum was necessary, and we could secure the patient’s postoperative LVEDV. A comparison of pre- and post-CT scans in both cases, showed increased LVEDV, especially in the first one.

Moreover, poor visibility caused considerable difficulty in the first case (Table 1). Therefore, we could not determine the correct direction or depth from the apex for the incision. Although this depends on the surgeon’s skills and experience, adding a transaortic incision and using a finger as a guide from the LVOT can be helpful. Intraoperative TEE is also beneficial.

**Table 1:** Comparison of the two aHCM and LVOTO cases

	aHCM	LVOTO
Cause of decreasing SV	LVDEV↓	LVOTS, SAM
Difficulties of procedure	Invisible	More visible
Surgical complications	Mitral valve injury, ventricular septal perforation, left coronary injury	Aortic valve injury, ventricular septal perforation

aHCM, apical hypertrophic cardiomyopathy; LVEDV, left ventricular end-diastolic volume; LVOTO, left ventricular outflow tract obstruction; LVOTS, left ventricular outflow tract stenosis; SAM, systolic anterior motion; SV, stroke volume

After a discussion with our team of cardiovascular surgeons and cardiologists, we reached a consensus to perform these procedures rather than provide medical treatment for the patients of the two cases. Consequently, we hope that the positive outcomes from these two cases will lead to improvements in extended myectomy.

Apical myectomies are rarely performed; however, there is a need to standardize this method. In Japan, heart transplantation for aHCM is not feasible because there are very few transplantation facilities. Heart transplantation is a longer procedure than extended myectomy. The latter can be challenging, but a detailed surgical procedure has been recently reported [3, 11–13]. Almost half of the global aHCM cases occur in Asian countries; therefore, clinicians should gather sufficient surgical experience in extended myectomy.

## Data Availability

All data generated or analyzed during this study are included in this published article. Data sharing is not applicable to this article as no datasets were generated or analyzed during the current study.

## Abbreviations

AML: Anterior mitral leaflet
CT: Computed tomography
aHCM: Apical hypertrophic cardiomyopathy
ICD: Implantable cardioverter-defibrillator
LV: Left ventricular
LVEDV: Left ventricular end-diastolic volume
LVOTO: Left ventricular outflow tract obstruction
LVOT: Left ventricular outflow tract
LVOTS: Left ventricular outflow tract stenosis
SAM: Systolic anterior motion
SV: Stroke volume
TEE: Transesophageal echocardiography
TTE: Transthoracic echocardiography
